# The SOC–COM-B Framework for Sustainable Food Choices: Tailoring Mobile Application Content to Consumers’ Behavioural Readiness

**DOI:** 10.3390/nu18213707

**Published:** 2026-11-08

**Authors:** Anna Rogala, Renata Nestorowicz

**Affiliations:** Department of Marketing Strategies, Institute of Marketing, Poznań University of Economics and Business (PUEB), Niepodległości Av. 10, 61-875 Poznań, Poland; renata.nestorowicz@ue.poznan.pl

**Keywords:** mobile applications, sustainable food consumption, healthy eating, nutrition apps, food choice, consumer behaviour, stages of change, COM-B, behavioural interventions, food literacy, digital food tracking

## Abstract

Background/Objectives: Consumers often hold positive attitudes towards sustainable food, but this rarely translates into behaviour, as everyday food decisions are shaped by routines, constraints and situations. Research on food-related mobile applications has focused mainly on their functions or persuasive power, with less attention to whether their content matches the conditions under which consumers decide. We conceptualise such applications as decision-support environments and examine how their content could be tailored to behavioural readiness to change. Methods: We combined the Transtheoretical Model (stages of change, SOC) and the COM-B model. Three quantitative studies among Polish consumers examined food literacy, decisional balance, values, well-being and use of mobile applications. Results: We found non-linear differences across stages in motivational profiles and values, with health as the most consistent motivator. Readiness was not linearly related to well-being or engagement with digital tools: consumers at the earliest stage did not report the lowest well-being, and some consumers willing to use sustainability-oriented applications were unaware that such applications existed. Conclusions: Standardised, persuasive approaches are, therefore, of limited use. We propose the SOC–COM-B framework as a conceptual and decision-support design approach for organising application content according to behavioural readiness. It is a basis for prospective testing and informs developers of nutrition-related applications.

## 1. Introduction

Mobile applications have become an important interface for consumers’ everyday decisions, acting as a two-way communication channel between customers and retailers. Mobile commerce is characterised by interactions inclined to interruptions and distracted attention. This is because mobile shopping usually takes place during everyday activities, rather than long, consistent, focused sessions [1]. Consumers often use mobile apps, routinise this usage, and integrate them into their daily practices [2,3]. When making decisions, consumers do not continuously and carefully consider the different options, especially in low-engagement and repeated decisions. Instead, they rely more on their habits and simplified cues [4]. We argue that application interfaces organise access to information, evaluation cues, and choice options, often under conditions of limited user attention. As such, applications serve not only as transaction platforms but also as environments that support sustainable decisions.

Public awareness of the environmental and health effects of food consumption is increasing, as is interest in sustainable practices [5,6]. Sustainable and healthy diets are, in practice, closely linked: frameworks such as the EAT–Lancet Commission’s planetary health diet and the FAO/WHO guiding principles for sustainable healthy diets treat environmental sustainability and nutritional adequacy as complementary rather than competing goals [7,8,9,10]. Individuals’ lifestyles and value systems have also changed [11]. Research on sustainable food consumption has advanced considerably. However, less attention has been paid to how digital environments structure everyday food-related decisions. Sustainable food choices are often not single decisions, but develop through daily practices, such as regular shopping and meal planning. Studies show that many food-related decisions are made habitually and with limited deliberation, even when they involve sustainability-related attributes, such as organic production or ethical sourcing [12,13]. At the same time, sustainability introduces additional informational and value-based demands—including labels, certifications and ethical claims—that must be processed by consumers alongside price, taste, convenience and established habits [14,15]. Sustainable food choices, therefore, combine high decision frequency with increased cognitive and evaluative complexity.

Research consistently shows a persistent attitude–behaviour gap in sustainable food consumption [13,16,17,18]. Factors such as food prices, limited availability, workday organisation and culturally embedded food preferences shape the conditions under which food choices are made, and often constrain the integration of sustainability considerations into routine decisions [19]. Social-psychological models, such as the Transtheoretical Model (TTM), have been widely used to explain these dynamics, but existing research has primarily focused on identifying behavioural stages or actions to be achieved [20,21]. To our knowledge, little attention has been paid to how heterogeneous readiness for change, the ability to process information, and decision-making tensions affect the organisation of everyday decision-making environments.

Mobile applications supporting sustainable food consumption have mainly been analysed in terms of specific application features, content elements or choice cues, and their associations with attitudes, intentions, and self-reported behavioural outcomes [22,23,24,25]. They often assume that users are relatively undifferentiated and, therefore, have homogeneous needs and expectations regarding an application’s features and content. When mobile applications produce insufficient sustainability-oriented outcomes, this is often interpreted as evidence of weak motivation or low user engagement. Such outcomes may instead result from a mismatch between the design of the mobile decision-making environment and the heterogeneous conditions in which users make food-related decisions.

In this paper, we examine how behavioural readiness for sustainable food consumption differentiates the decision-making conditions required within a mobile application that supports such consumption. Insofar as mobile applications increasingly serve as informal channels of nutrition-related education and health promotion, understanding how their content should be organised is also relevant to broader efforts to promote healthy eating.

Our study contributes to research in e-commerce and digital marketing that conceptualises marketing as the management of information, signals and decision-making environments in which consumers operate. Within this approach, companies are seen not only as triggering change through persuasive tools, but also as shaping and configuring the informational and structural conditions under which everyday decisions are made.

## 2. Theoretical Background

This section argues that digital and mobile decision-making environments should be analysed not only in terms of technological affordances, but primarily in terms of the alignment between content structure and consumers’ situational behavioural readiness.

### 2.1. Decision-Making Conditions in Digital and Mobile Environments

In recent years, online shopping has grown rapidly worldwide, significantly transforming how consumers make purchasing decisions [26,27]. E-commerce platforms allow users to view all products and related information to support their decision-making by minimising doubts and building the initial trust needed for online purchasing [28,29]. Software technology is used to understand consumers’ needs, expectations, and preferences, and to provide useful recommendations and information [28,30]. As a result, digital environments not only transmit information; they structure access to it, shape the sequence of actions, and expose specific options and constraints. With their precise design and programming, these environments influence both cognitive and behavioural outcomes. They function as active structures that organise visibility, attention, and behaviour instead of being passive transmission channels [31].

Decision-making in digital environments is conditioned by numerous internal and external factors [32], and consumer needs, expectations, and decision strategies differ across product categories, decision types, and contexts [33,34]. This is in line with prior research showing that consumer decisions are contextual and shaped by situational factors [33,34]. In e-commerce and m-commerce settings, choices are mediated by the environment in which they are made. Choices change systematically, driven by information structure, digital context and task architecture.

A growing challenge associated with online decision-making is information overload [22,35,36,37], which can frustrate users, distract them, and trigger context-dependent routines that change how they make decisions. Therefore, addressing this challenge when designing a decision platform and its features is critical [38], particularly given the limited research offering an integrated view of how multiple aspects of online decision-making interact [39]. These limitations are more pronounced on mobile devices, because people have less cognitive, attentional, and temporal resources.

Mobile applications differ from other digital platforms in terms of their usage context, interface characteristics and integration with everyday consumption practices. Decision-making on these platforms tends to be fragmented, occurring in short, frequent sessions. It is also limited by the available space for information. Repeated interruptions and context-dependent interaction styles influence the efficiency with which consumers process information and make decisions [40]. Consequently, mobile shopping reflects situational, on-the-go usage patterns that differ essentially from more deliberative desktop-based browsing [37].

Although these characteristics are well documented, much existing research continues to conceptualise mobile applications primarily as technological tools or persuasive devices [41,42,43]. Other research has focused on system design, technological functionalities, and mechanisms of adoption [44,45]. Relatively limited attention has been paid to how the structure and content organisation match consumers’ situational capacity and willingness to process information [46]. This is especially evident in complex decision-making scenarios, such as sustainability-oriented consumption, where trade-offs are present.

The above points show that we need research based on a theoretical perspective that links digital environments with the situations consumers are in [39,47]. We, therefore, conceptualise digital and mobile decision-making as the outcome of interactions among content structure, context of use and behavioural readiness. This establishes the foundation for the more focused conceptual developments that follow.

### 2.2. Sustainability as a Decision Context in Food Decisions

From a decision-making perspective, food choices are characterised by short, passive, low-effort, and low-involvement processes that are automatically triggered by contextual factors, routines, cues, and norms [2,48,49]. Sustainable food choices are made on a similar basis [22,23,50]. Food decisions are influenced by habits, environmental signalling, and time pressures, and the decision-making process is not separate or deliberate [22,23,50]. The routine-based, low-involvement nature of these choices presents a specific challenge for digital decision support. This is because they are shaped more by situational cues and environmental factors than by careful evaluation.

Research indicates that a positive attitude towards sustainability can influence food choices, but these factors are often less important than taste, price, health, and convenience [5]. Different types of customers have different priorities [51]. While many consumers consider sustainability-related factors alongside others, such as origin, fair trade labelling, and food category, these do not replace their primary motivations. Such trade-offs are part of routine consumption and reflect the tensions that consumers manage or try to resolve. Therefore, we treat sustainability as an additional dimension when evaluating food options.

Mobile applications are increasingly influencing key areas of food-related decision-making, such as purchasing, meal planning, and consumption. They have a greater impact on immediate decision-making than individual preferences or motivations [52,53,54,55,56]. Although more information about sustainability is available, this availability alone has not been sufficient to influence daily food choices [3]. The limited success of information-based approaches indicates that obstacles to sustainable food choices are rooted more in environmental and structural factors that shape daily decisions than in consumers’ motivation or values. This emphasises the significance of the decision environment as an area of analysis [57].

We argue that the sustainability decision context consistently creates tensions, trade-offs, and ambivalence, and highlights value conflicts rather than a lack of motivation. These features create a context in which sustainability-focused food choices are likely to be misaligned with the content and functionalities of the standardised digital tools that should enable them. This is the result of differentiated decision-making circumstances.

### 2.3. Behavioural Readiness and Decisional States Shaped by Situational Context

Previous studies on behaviour change indicate that having appropriate knowledge is not enough to motivate action [58,59,60,61,62,63]. Behavioural outcomes depend on individuals’ readiness to engage in change, personal assessment of benefits and costs (decisional balance), and confidence in the ability to act. The Transtheoretical Model (TTM) of Behaviour Change, developed by Prochaska and DiClemente, draws on multiple psychological theories to identify the processes that facilitate change within a specific behavioural domain [64]. It has already been effectively applied in interventions that encourage sustainable behaviours [65]. In the present study, the TTM is employed as an analytical framework to distinguish different decisional states, rather than as a descriptive model of behavioural progression.

According to the TTM, behaviour change consists of five constructs: stages of change, processes of change, decisional balance, self-efficacy, and temptation [66]. In this study, we focus on two of them—stages of change (SOC) and decisional balance (DB)—as they directly underpin both the empirical design and the proposed SOC–COM-B framework. The five stages are [58,65,66,67]:

1. Precontemplation—no intention to change;
2. Contemplation—awareness and consideration of change;
3. Preparation—intention and initial steps towards change;
4. Action—recent adoption of the behaviour;
5. Maintenance—sustained performance of the behaviour over time.

Progression through these stages is not linear, and individuals may move back and forth between stages [21,67]. Importantly, an individual’s stage of change has been shown to predict subsequent behaviour [65,66]. We conceptualise stages of change not as a goal-oriented sequence of desired transitions, but as an analytical framework that captures qualitatively distinct decisional states in which consumers engage with information and make choices.

Research indicates that progressing to the action stage requires a marked increase in perceived benefits. In precontemplation, cons outweigh pros. In later stages, there is a reverse dependency, with the shift occurring before action. Early-stage interventions should highlight benefits, whereas later-stage strategies should minimise disadvantages. High levels of both pros and cons suggest ambivalence [65,68]. A similar pattern was observed across dietary changes [69].

Furthermore, prior research suggests that different decisional states are associated with differences in how individuals process information [67,69]. Cognitive and affective processes tend to dominate in earlier stages (precontemplation and contemplation). Action-oriented processes become more dominant in later stages (preparation, action, and maintenance). Importantly, the relevance of these processes differs across behaviours and contexts [65].

These assumptions mean that the TTM is particularly good at designing digital interventions, because the same information or content can have different effects depending on users’ behavioural readiness. This suggests that the content of mobile applications, such as information, arguments, and cues, is likely to be interpreted differently by different users and does not guarantee the same effects for everyone. Interpretation depends on users’ key decisional tensions and perceived pros and cons.

In our study, behavioural readiness is not viewed as a point on a linear progress scale but as a specific situation that comprises ability, attention, and experienced tension. Therefore, people can exist in different qualitative readiness states that influence how they notice, interpret, and respond to information, without necessarily moving towards a specific behavioural goal. In this approach, differentiated readiness is a source of unique information-processing conditions. That is why the same content can be perceived as irrelevant, demanding, motivating, or actionable, depending on how cognitive capacity, focus, and decision-related tensions are set in each situation.

### 2.4. Content Structure as an Element of Decision-Support Environments

A positive attitude towards sustainable products is a necessary but insufficient condition for sustainable consumption: a persistent gap separates what consumers declare from how they actually behave [13]. The literature names this inconsistency in several ways—the attitude–behaviour gap, the intention–behaviour gap, the motivation–behaviour gap, the value–behaviour gap [17,70,71,72]. If attitudes are already in place, interventions that work on attitudes address a condition that has already been met, and the leverage must lie elsewhere: in the conditions under which everyday decisions are made.

Where exactly it lies has been studied extensively [6,15,73,74,75,76]. Habits, competing motivations, situational factors, such as promotions, and individual characteristics, including values, knowledge and perceived behavioural control, all contribute to the gap [76]. The attitude–behaviour–context (ABC) theory locates the mechanism in the interaction between the two: behaviour follows from attitudes and context together [77], which means that a sufficiently unsupportive context suppresses behaviour no matter how favourable the attitude. Research on the value–action gap reports this pattern, with pro-environmental attitudes failing to translate into action where contextual and routine-based factors work against them [3]. Attitudes, in other words, need contextual reinforcement to become behaviour [78,79]—and this is what makes the context of an intervention, including the digital environment in which it is delivered, a design variable rather than a background condition.

Digital tools supporting sustainable food practices range from simple tracking and information-based applications to more advanced interactive solutions [80]. Across this range, the influence on everyday food decisions is exerted less through what the tool can do than through how its content is structured. Informational cues, stimuli, feedback, and guidance embedded in routine food practices determine what users notice, how much cognitive effort a choice requires, and which options are visible at the moment of choice. Content, in this reading, is not only a means of providing information. It is an organising structure that mediates decisions taken under limited attention, time pressure and habit.

This suggests that digital tools function as environments that support decision-making, and that their effect depends on how content is designed and presented. The same content will not work the same way for every user: it interacts with individuals’ decisional states and situational conditions. We, therefore, examine how content may support sustainable food choices for some consumers while constraining them for others.

### 2.5. Overcoming the Attitude–Behaviour Gap in Sustainable Food Consumption Through the Use of Digital Tools

We argue that digital tools can help bridge the gap between attitudes towards sustainable consumption and actual behaviour, provided their design accounts for differences in behavioural readiness and in the conditions under which decisions are made. The COM-B framework, in which behaviour results from interactions among capability, opportunity, and motivation, is well suited to this purpose: it translates abstract behavioural barriers into operational requirements that content and application functionality can address [81]. This matters because motivational appeals dominate communication practice, on the assumption that they trigger the desired behaviour [80]. Yet motivation is often not what is missing. Consumers who already want to eat more sustainably still lack the capability to break established habits and the opportunity to find sustainable options where they actually shop. We, therefore, integrate COM-B with the food environment perspective and identify six conditions for sustainable consumption: consumer capability, motivation, and opportunity, together with the attractiveness, affordability, and accessibility of sustainable food options [82].

The TTM and COM-B answer different questions, which is why we use both. Stages of behavioural readiness indicate when a consumer is open to support; COM-B specifies what that support must supply—capability, opportunity or motivation—at each stage. Designing effective stage-matched digital support, therefore, requires distinguishing consumers by stage of change, identifying the barriers and enablers relevant to each stage, and addressing them through appropriate content and functional support. Evidence from health, dietary and pro-environmental domains indicates that such approaches can sustain behaviour change over time [65].

What determines the effectiveness of these tools is rarely technological sophistication [80,83]. It is whether they support self-monitoring, make consumption patterns visible, and deliver timely, actionable feedback at the moment a decision is made [80,83]. Smartphone applications are frequently compared with traditional interventions [25,84,85] because they are accessible, widely used and relatively inexpensive [86]. Their potential to shape everyday food choices remains largely unrealised, and much of that potential lies in content rather than in features: the informational cues, feedback and guidance that become part of everyday food practices. Behavioural models matter here precisely because they explain why the same content changes behaviour for one user and passes unnoticed by another.

Sustainable food consumption is limited by both practical and psychological barriers. Mobile applications can address some of them by providing practical information and habit-relevant tools, yet their effectiveness is constrained by perceived complexity, time pressure and declining engagement over time. Research identifies such barriers at every stage of the consumer journey [24,86,87,88], and points to the need for content and interaction flows that fit users’ daily routines rather than adding to their mental or time burden. This matters all the more because sustainability is rarely the primary reason for using an application. Applications of this kind operate through opportunity and capability rather than motivation, and they can reach users who would not respond to a sustainability appeal at all but would use them for convenience or profit.

Prior studies link digital tools to sustainable consumption, but, to our knowledge, little is known about how behavioural readiness shapes the kind of content and support that consumers actually require. Stage-based readiness (SOC) and COM-B mechanisms are seldom brought together in work on digital sustainability interventions. The omission is consequential in mobile contexts, where content mediates food-related decisions taken under limited attention and heavy habit.

This study, therefore, examines how differences in SOC translate into distinct behavioural, motivational, and value-based profiles, and how those profiles could inform content in sustainable-food mobile applications. The question is not whether such applications are adopted or effective in general terms; it is what different consumers need from them. The profiles serve as the basis for a consumer-first, content-oriented framework that integrates SOC segmentation with COM-B components.

The contribution of the SOC–COM-B framework lies in combining two questions that are usually addressed separately. Stage-based interventions specify when a consumer is open to support [58,59,83], whereas COM-B-informed approaches specify what support must be supplied [81,83]. Existing digital food-choice support tends to treat users as undifferentiated or to concentrate on features and persuasive cues [22,23,24,25,41,42,43]. Here, behavioural readiness is used to structure the content of the application, and COM-B to name the type of support that content has to provide. The framework is proposed as a conceptual and decision-support design approach, i.e., a way of organising content and support functions in application design. It is not a diagnostic instrument for identifying individual users’ needs, and its validity as a measurement tool has not been established.

We address the following research questions:

- RQ1: How do consumers at different stages of change differ in terms of behavioural, motivational, and value-based characteristics that are important for content design in sustainable-food mobile applications?
- RQ2: How can these differences be translated into a stage-based framework for the design of content and support mechanisms in mobile applications oriented towards sustainable food consumption?

The analyses draw on three quantitative studies. Together they provide the empirical basis for a framework that proposes how mobile applications can build capability, create opportunity, and stimulate motivation through stage-matched content and functional support, rather than through standardised persuasion. Understanding what SOC groups share and where they diverge is what makes it possible to guide content, communication style and application functionality towards consumers’ actual behavioural readiness.

## 3. Materials and Methods

To answer the research questions, three quantitative studies were conducted with Polish consumers. The studies were designed to identify patterns and differences in behavioural readiness and related profiles across the SOC groups. It is important to note that we did not aim to assess the effectiveness of specific interventions or applications.

Each study provided empirical data to understand differences in behavioural readiness across stages of change in the context of sustainable food consumption. The three studies were aligned in scope and, in part, in measurement structure (Study 3 used a different operationalisation of behavioural readiness, see below), enabling an integrative interpretation of behavioural readiness across groups, even though they were conducted at different time scopes. The use of three studies enables a more reliable identification of SOC-related patterns across different contextual and temporal conditions, while maintaining a consistent conceptual framework.

Sustainable food behaviours can be exhibited by choosing plant-based products more often than animal-based ones, selecting products from local suppliers, choosing seasonal vegetables and fruits, and choosing products from ethical farming systems (e.g., free-range eggs), free from antibiotics and hormones, and certified products (e.g., organic or fair trade). They also include practices to reduce food waste, such as planning shopping trips, using ingredients efficiently, and sharing excess food. This operationalisation reflects a broad, practice-oriented understanding of sustainable food consumption that extends beyond specific dietary choices.

The Transtheoretical Model (TTM) Readiness to Change scale proposed by Prochaska and DiClemente [64] was used to classify respondents in Studies 1 and 2 by stage of change towards sustainable food behaviours. The SOC classification was used to analyse different decisional states associated with sustainable food practices, not as an indicator of linear behavioural progression.

The stage of change was assigned using the TTM Readiness to Change scale [63,64,65,68]. The scale s used to classify stages for dietary and pro-environmental behaviours [89]. We did not test the measurement invariance of the scale across studies and the treated SOC as a pragmatic grouping variable and not as a validated psychometric construct in the studied samples.

In Studies 1 and 2, the respondents were given the following definition of sustainable dietary behaviour: ‘Sustainable dietary behaviour involves choosing plant-based products more often than animal-based ones; products from local suppliers; seasonal fruit and vegetables; and meat, fish and eggs from ethical farms where animal welfare is prioritised, such as free-range eggs; products that are free from antibiotics and hormones; and certified products, such as organic food and fair trade. It also involves reducing food waste and sharing food.’ They were then asked to rate their own behaviour on a scale: 1—I do not think about it at all; 2—I think about it, but I am not convinced; 3—I have decided to do so and am now thinking about how to start; 4—I am trying to do so, but it is difficult; or 5—I do this regularly/every day. The same scale was used in Study 3, but this time the respondents used it to describe their behaviour in relation to specific aspects of sustainable eating habits. A total of 15 statements were evaluated: (1) “When I go shopping, I use a list I’ve prepared in advance”; (2) “I know how much and what kind of food I have at home”; (3) “I’m reducing my meat consumption”; (4) “I’m reducing my consumption of animal-based products, such as dairy and eggs”; (5) “I eat meat, fish and eggs sourced primarily from ethical farms where animal welfare is a priority”; (6) “I buy my food mainly from local suppliers and farmers”; (7) “When shopping, I mainly choose seasonal fruit and vegetables”; (8) “I buy products with a short use-by date”; (9) “I only buy as much food as I can eat before it expires or goes off”; (10) “I buy certified products (e.g., organic and fair trade food)”; (11) “I buy food without unnecessary packaging or in environmentally friendly packaging”; (12) “If I have a product whose use-by date is approaching, I use it to prepare a meal”; (13) “If I have a product whose use-by date has passed but which haven’t gone off, I use it to prepare a meal”; (14) “I store and use up leftover food”; and (15) “I share food when I have too much of it”. The statements were developed by the authors on the basis of a review of the literature on sustainable food behaviours [90,91,92,93] and adapted to the Polish context; they were inspired by, but do not reproduce, existing scales measuring these behaviours. To determine the level of sustainable food consumption, we calculated the average score across all statements. These values were then used to divide the respondents into five quintiles of increasing reported behavioural change (BRG). We refer to these as behavioural-readiness groups (BRG1–BRG5). They are ordinal, sample-relative categories and are not equivalent to the theoretically defined stages of the Transtheoretical Model; we, therefore, do not compare them with the SOC groups of Studies 1 and 2. Study 3 was used to examine whether value orientations and decisional balance vary along a gradient of reported behaviour. In this sense, it complements, rather than replicates, Studies 1 and 2, and it contributes to the framework only at the level of conceptual interpretation.

The Cronbach’s alpha value was 0.829, indicating very good internal consistency. External validity is limited by reliance on self-reported change in a quota sample of Polish internet users (see Section 5.3). Study 1 examined how differences in SOC were associated with respondents’ engagement with food-related mobile applications, alongside food literacy levels measured using the Short Food Literacy Questionnaire (SFLQ) [94]. Measures of food-related mobile applications were included as descriptors of decision-support use in everyday food practices, not as indicators of technology adoption or intervention outcomes.

Studies 1 and 3 employed the Decisional Balance (DB) scale to assess perceived advantages and disadvantages of adopting more sustainable eating practices [89]. Studies 1 and 2 assessed satisfaction with food-related life using the SWFL scale [95]. In addition, Study 2 included a measure of general technology acceptance based on Rogers’ diffusion framework, while Study 3 focused on the relationship between consumers’ value orientations (PVQ-RR questionnaire identifying 19 values) [96] and behavioural readiness (BRG).

The statistically significant results are presented in the main text; the results of the remaining analyses are provided in Supplementary Materials in Tables S1–S4. Effect sizes (Cramér’s V for χ^2^ tests and η^2^ for ANOVA) are reported together with *p*-values. Hochberg’s GT2 and Games–Howell tests control the family-wise error rate for pairwise comparisons within each variable. For the value rankings, the ratings of the 19 PVQ-RR values were ranked, and the mean rank of each value was calculated within each group; the values in parentheses in Table 6 are these mean ranks. Differences in the mean ranks were tested with Friedman’s test, and pairwise differences with the Wilcoxon signed-rank test. The detailed results of the Friedman and Wilcoxon tests are presented in Tables S2 and S3.

### 3.1. Characteristics of Respondents

The studies were conducted using commercial online access research panels of professional research agencies (Biostat for Studies 1 and 2, and Ariadna for Study 3), in accordance with a research plan agreed between the authors and the agencies. The samples were drawn using quota sampling. In non-probability panel research, the sampling frame and the number of invited persons are not defined in the way required for calculating a classical response rate; we, therefore, describe the sampling procedure, the quota structure (Table 1), and the quality-control procedures instead, and we do not claim statistical representativeness of the samples. The research agencies adhere to the ICC/ESOMAR International Code on Market, Opinion and Social Research and Data Analytics and applicable data protection regulations, including the General Data Protection Regulation (GDPR). They implement systematic quality assurance procedures throughout data collection, including monitoring questionnaire completion times, analysing response patterns and logical consistency, and using attention checks to identify insufficient respondent engagement. Additional safeguards include verifying respondents’ exposure to audio-visual research stimuli, applying participation restrictions to prevent excessive or repeated involvement in surveys, and rechecking eligibility and sample quota criteria. Furthermore, controlled panel recruitment practices are used to ensure transparency regarding respondent sources and minimise the risks associated with uncontrolled recruitment channels. Collectively, these measures help safeguard sample integrity and reduce the likelihood of unreliable or low-quality responses being included in the final dataset. Respondents completed the questionnaire using a web application or a mobile app available on Android and iOS devices.

The final samples comprised 600 (Study 1), 600 (Study 2), and 639 (Study 3) respondents who completed the questionnaire. Quota sampling was applied based on gender, age, and place of residence, so that the sample structure approximated that of Polish internet users aged 18–54 in Study 1, and 18–59 in Studies 2 and 3. Data collection was conducted in 2021, 2022, and 2024. Detailed characteristics of the respondents are presented in Table 1.

Table 2 shows the structure of samples based on the declared achievement of subsequent levels (SOC) of sustainable food behaviours.

Study 1 was conducted in 2021, during the COVID-19 pandemic, which constituted a specific contextual condition at the time of data collection. For this reason, we do not compare the sizes of the groups achieving the various SOCs across the two studies.

The results were analysed with SPSS Statistics 29.0 for Windows. The following tests and methods of statistical analysis were used: Student’s t-test, χ^2^ test, Friedman’s test, Wilcoxon signed-rank test, and one-way Analysis of Variance (ANOVA). For data that met the assumption of homogeneity of variance (based on Levene’s test) [99], we applied the post hoc Hochberg’s GT2 test [100]; for data that did not meet this assumption, the Games-Howell test was applied [101]. All statistical analyses were selected to examine group-level differences across SOC categories in line with the research questions, rather than to test causal relationships.

### 3.2. Operationalisation: Assigning Variables to COM-B Domains 

To make the link between the data and the framework transparent, Table 3 maps the measured variables onto the COM-B domains.

### 3.3. The Development of the Framework and Levels of Evidence

The framework was developed in three steps. First, we listed the statistically significant differences across groups, as shown in Table 4, Table 5 and Table 6 (food literacy, decisional balance, values, and awareness of and interest in applications). Second, we assigned each difference to the COM-B domain to which it is conceptually closest (Table 3). Third, we formulated, for each group, the implications for content and support that follow from the theoretical interpretation of that difference. Table 7 and Table 8 are not results of statistical analysis,, therefore, they should be read as design hypotheses and not as tested requirements.

Throughout the manuscript, we distinguish three levels of evidence. (1) Empirically observed differences across groups of different behavioural readiness (Section 4.1 and Section 4.2, Table 4, Table 5 and Table 6). (2) Conceptual interpretations resulting from the integration of the SOC and COM-B (Section 4.3 and Section 5). (3) Proposed application-design strategies, which are formulated as hypotheses and require prospective validation (Table 7 and Table 8). The studies were not designed to test the effectiveness of any application content; statements about what content may be needed should, therefore, be read as design hypotheses derived from levels (1) and (2).

## 4. Results

The results show behavioural and motivational differences across the SOC groups. Higher SOC levels are associated with greater food literacy, food well-being, and declared engagement with sustainability-related mobile applications. Accordingly, the results are presented as comparative profiles across the SOC groups. The differences are observational and are not interpreted causally.

### 4.1. SOC Differences in Engagement with Food-Related Mobile Applications

Individuals across the different SOCs differed in demographic characteristics, such as gender and education. Women constituted a larger proportion at the final stages of behaviour change, while men dominated at SOC1 (Table 4). Educational attainment also differed across the SOC groups. In the action and maintenance groups, at least half of the individuals had higher education, whereas in the precontemplation group, fewer than 30% did. Apart from gender and education (in the first two studies), no other demographic features significantly distinguished the respondent groups. The highest levels of food literacy were observed among the respondents in SOC4 and SOC5. Across Studies 1 and 2, food-related well-being (FWB) increased with higher SOC levels and reached its highest values in the maintenance group. Notably, respondents in the precontemplation stage did not report the lowest levels of FWB, despite lower food literacy scores. Gender and education differed across SOC groups (Table 4). We treat them as characteristics of the groups, rather than as nuisance variables; the comparisons were not adjusted for them, so differences in food literacy, well-being or application use may partly reflect group composition.

No statistically significant differences were observed across the SOC groups in their general attitudes towards technological innovations (Study 2). Across all groups, respondents displayed moderate, comparable levels of technology acceptance, with no group disproportionately represented as either early adopters or technology-averse users. Table 5 presents data on food-related mobile applications, categorised according to their primary functions: providing product-related information (e.g., ingredients and product features), supporting food-waste reduction, and enabling lower-priced food purchases. This functional categorisation was applied consistently in both Study 1 and Study 2.

The level of familiarity with food-related mobile applications varied across the SOC groups. Regardless of the application type, the respondents in the precontemplation stage reported the lowest levels of familiarity and interest in using such applications. Within this group, relatively higher interest was observed for applications enabling lower-priced food purchases compared to the other two application categories. Low awareness of food-related applications was also observed among the respondents in higher SOC groups, despite their declared willingness to use them, particularly in the action stage. Reported use or intention to use applications was more prevalent among the respondents in the preparation, action, and maintenance stages than among those in earlier stages.

The analysis of Decisional Balance (DB) revealed systematic differences across the SOC groups. The respondents in the two highest SOC stages expressed the most positive evaluations of the advantages (PROS) of adopting sustainable food behaviours, whereas those in the precontemplation stage reported the least positive evaluations, across both studies (for Study 3: behavioural-readiness groups). In contrast, the respondents in the precontemplation stage did not report the highest levels of perceived disadvantages (CONS). Higher levels of critical evaluation were observed among the respondents in intermediate SOC stages, while respondents in the maintenance stage reported the lowest levels of perceived disadvantages. Among the potential PROSs, the respondents in each group most frequently cited factors related to the positive health impacts of sustainable consumption. Among the CONS, the most frequently cited issues concerned the high cost of such behaviours, regardless of the SOC achieved.

### 4.2. Value Orientations Across Behavioural-Readiness Groups

To examine differences in value orientations across behavioural-readiness groups (BRG1–BRG5; Study 3), the values were ranked within each group, and the statistical significance of differences among individual values was tested. The most important results are presented in Table 6.

Across all behavioural-readiness groups, societal security and self-direction–action consistently ranked as the most important values. Benevolence–dependability was also among the top three values in most groups. In contrast, in the BRG2 group, benevolence–dependability did not appear among the three highest-ranked values and differed significantly from the two leading values.

Across all groups, the lowest-ranked values were power–dominance, power–resources, and stimulation. Differences across groups were also observed for selected values, particularly universalism–nature, hedonism, and tradition. The importance of universalism–nature values increased with higher readiness levels. Hedonism and tradition were relatively more noticeable among the respondents at lower readiness levels.

### 4.3. SOC-Matched Content and Decision Support in Sustainable Food Consumption Applications

The similarities and differences observed across the SOC groups suggest stage-specific design hypotheses for the communication approach and functional focus of applications supporting sustainable food consumption (Table 7). These design hypotheses are derived from systematic differences in behavioural readiness, decisional balance and value orientations. They are not based on consumers’ stated preferences for application content, and their effectiveness has not been tested.

Declared interest in applications is highest in SOC2–SOC4, among the respondents who are actively deciding about changes to their food behaviour or already adopting new practices. In Study 1, actual use is highest in the maintenance stage (24–29% depending on the function), so the lower interest declared in this group partly reflects that many of these respondents already use the applications. The respondents in the precontemplation stage reported the lowest familiarity and interest.

The content that may be needed also appears to differ across groups. Value-aligned framing and the pros and cons that are relevant at a given stage may matter more than standardised sustainability messaging. Perceived benefits and barriers shift in relative importance across levels of readiness, and it is this shifting weight that may determine whether digital content is noticed and taken into account at all.

The factors examined in this study provide an analytical basis for understanding how such applications might support individuals at different stages of change across the three COM-B domains. Differences in food literacy, value orientations and decisional balance suggest that capability-related support may depend on matching the complexity of content to what users actually know and to how confident they are that they can change their behaviour.

In the opportunity domain, the results support conceptualising applications as environments that surface stage-specific benefits and barriers at the moments when food decisions are actually made. In the motivation domain, the differences in what matters to users at each stage suggest that content may need to speak to their current readiness rather than apply a standardised persuasive strategy.

The Discussion section develops this synthesis into the SOC–COM-B framework for designing mobile applications that support sustainable food behaviours. The framework consolidates the findings of all three studies into a single conceptual approach linking behavioural readiness to design hypotheses for content and functional support. 

## 5. Discussion

### 5.1. Main Findings

Engagement with application content appears to be driven by practical needs rather than ideological commitment. Across the SOC groups, interest in applications tracks specific functionalities—product information, food waste reduction, and cost-related features—more closely than sustainability messaging. This does not make values irrelevant. Value priorities differ systematically across stages, and benevolence–dependability, societal security and self-direction–action emerge as stage-relevant drivers of how content is interpreted and evaluated. Motivational cues, therefore, may not be standardisable: the same appeal may work differently depending on which values are relevant at a given stage.

Importantly, behavioural readiness is not linearly associated with either well-being or engagement with digital tools. The respondents in the precontemplation stage did not report the lowest levels of food-related well-being. This pattern suggests that food-related well-being does not increase linearly with behavioural readiness and may be shaped by separate decisional dynamics across the SOC groups. The relatively high well-being reported at SOC1 may have a practical consequence: consumers who are already satisfied with the way they eat have little reason to change, and appeals that presuppose dissatisfaction may not reach them.

These findings reinforce the significance of decisional balance. This is a key mechanism that shapes how content is processed by different SOC groups. Analysing the perceived advantages and disadvantages of sustainable food practices also provides further evidence of non-linearity. The respondents in intermediate stages of the SOC were more critical when evaluating the pros and cons. This reflects heightened decisional tension, whereas those in the maintenance stage reported the fewest perceived disadvantages. This suggests that SOC2–SOC3 are characterised by heightened decisional tension and implementation needs.

Engagement with food-related mobile applications is not simply a function of technological acceptance. General attitudes towards technology were similar across the SOC groups, yet awareness and use of sustainability-oriented applications varied considerably. The respondents in the action stage reported limited awareness of existing applications even while expressing willingness to use them—motivation and readiness were present, and the constraint lay elsewhere. Opportunity-related barriers, therefore, operate independently of both.

### 5.2. Interpretation Through the SOC–COM-B Framework

Supporting sustainable food consumption through digital tools may require a shift away from persuasion towards provision of support. Mobile applications are decision-support environments, not persuasive devices. We treat food-related applications as part of the digital retail infrastructure, supplying consumers with product information, pricing and purchase support.

These findings indicate that the TTM and COM-B can be integrated as complementary frameworks. The TTM supplies the temporal dimension—when a consumer is open to support—while COM-B specifies what that support must build: capability, opportunity, or motivation, through content and functional design. The integration may also change what behavioural models are used for. They can serve not only as segmentation instruments but also as an organising logic for content on digital platforms. For research on digital food and health applications, this proposes behavioural readiness as a principle for structuring content and interactions, complementing work on platform and application design that has concentrated on technological affordances, system architecture and user–technology interaction mechanisms [44,45,102,103].

Table 8 synthesises the empirical findings of the three studies and their translation into the stage-specific SOC–COM-B framework.

Several design guidelines hold across stages. Engagement depends on the perceived reliability and trustworthiness of the application and of the content it delivers. It also depends on simple, low-effort guidance that reduces uncertainty about how the application is to be used. Making use of the application visible to others, and supporting users’ confidence that they can learn it over time, further lowers entry barriers. These cross-stage guidelines complement the stage-based logic set out in Table 7.

The segmentation suggests that SOC2–SOC4 may be the group that matters most for sustainability-oriented applications: these consumers report high interest in practical features and greater receptiveness to personalised guidance. Food literacy and capability rise across stages, which suggests that the complexity of content could be varied accordingly—from simple, low-burden messages in the precontemplation stage to more advanced, data-rich information in the action and maintenance stages.

The findings also point to support-over-persuasion approaches to application design. We hypothesise that users at early stages may respond better to non-directive content that respects their autonomy and that, at later stages, they may become more receptive to goal-oriented and efficiency-focused support. Content could, therefore, be tailored to behavioural readiness rather than delivered through standardised persuasive techniques; this remains to be tested.

### 5.3. Limitations and Future Research

This study has several limitations. First, the data are observational and self-reported, so the associations cannot be interpreted causally and may be affected by social desirability. Second, the three studies differ in time (2021, 2022 and 2024; Study 1 was conducted during the COVID-19 pandemic), in the measures used, and in the way behavioural readiness was operationalised (SOC stages in Studies 1–2; quintile-based groups in Study 3); they were, therefore, not compared directly. Third, the samples come from an online panel and were drawn by quotas, so they are not probability samples. Fourth, all samples are from Poland, which limits generalisation to other food cultures and digital markets. Fifth, no application content was tested: the stage-to-content assignments in Table 7 and Table 8 are hypotheses that have not been tested with users or evaluated by application designers, and the framework is not derived from a validated intervention model, so it cannot be used to measure the effectiveness of interventions or to classify individual users. Sixth, gender and education were associated with readiness group, and the group comparisons were not adjusted for them; we, therefore, interpret the results at the group level and do not attribute the differences to readiness alone. Multivariate models are a task for future research.

Validation of the framework would require prospective designs. For example, experimental or longitudinal studies could compare stage-matched with non-stage-matched application content using predefined engagement outcomes (e.g., sustained use or feature use) and behavioural outcomes (e.g., change in the purchase or consumption of selected food categories). Such studies should also test whether the framework improves decisions beyond existing digital nutrition-support approaches, validate the classification of users before it is used for tailoring, and examine whether integrating further behavioural constructs refines the proposed intervention paths.

### 5.4. Contribution to Nutrition Research

The framework may be relevant to nutrition research because applications that support sustainable food choices also convey information on healthy eating, and health was the most consistent motivator across all groups in our data. Matching content to behavioural readiness could, therefore, inform future nutrition-related digital interventions, for example, by deciding whether an application should first provide basic information, support decisional balance, or help to maintain existing routines.

## 6. Conclusions

This study examined how behavioural readiness for sustainable food consumption, assessed in three studies among Polish consumers, differs in relation to food literacy, decisional balance, values and engagement with food-related applications. The differences were systematic and non-linear, and health was the most consistent motivator across groups. On this basis, we propose the SOC–COM-B framework as a conceptual and decision-support design approach for organising application content according to users’ behavioural readiness. The framework suggests that standardised persuasive content is likely to match the needs of only some users, and that capability- and opportunity-related support may be as important as motivational appeals. These design implications are hypotheses: they were not tested in this study, and the framework has not been validated as a diagnostic or measurement instrument. Prospective comparisons of stage-matched and non-stage-matched content are the next step.

## Figures and Tables

**Table 1:** The profile of the respondents.

Variable		Sample Frequency (%)		
	Population (Thousands)	Study 1 (2021)	Study 2 (2022)	Study 3 (2024)
Total	21,184.9 (100.0)	600 (100.0)	600 (100.0)	639 (100.0)
Gender				
female	10,520.9 (49.7)	283 (47.2)	298 (49.7)	325 (50.9)
male	10,664 (50.3)	317 (52.8)	302 (50.3)	312 (48.8)
not available	-	-	-	2 (0.3)
Age *				
18–29	4848 (22.9)	207 (34.5)	141 (23.5)	135 (21.1)
30–39	5920.9 (27.9)	203 (33.8)	168 (28.0)	188 (29.4)
40–49	5868.2 (27.7)	150 (25.0)	163 (27.2)	179 (28.0)
50–59	4547.8 (21.5)	40 (6.7)	128 (21.3)	137 (21.4)
Education **	20,623.5 (100.0)			
No education or unknown	709.7 (3.4)	-	-	-
Primary (P)	1373.4 (6.7)	15 (2.5)	10 (1.7)	9 (1.4)
Basic vocational (BV)	4256.5 (20.7)	56 (9.3)	76 (12.7)	54 (8.5)
Secondary (S)	7595.4 (36.8)	272 (45.3)	282 (47.0)	237 (37.1)
Higher (H)	6688.5 (32.4)	257 (42.8)	232 (38.7)	339 (53.1)
Place of residence ***	37,907.7 (100.0)			
Village	15,283.7 (40.3)	239 (39.8)	240 (40.0)	247 (38.7)
City up to 20,000 inhabitants	4941.1 (13.0)	78 (13.0)	78 (13.0)	82 (12.8)
City from 20,000 to 100,000 inhabitants	7126.4 (18.8)	115 (19.2)	115 (19.2)	123 (19.2)
City over 100,000 inhabitants	10,556.5 (27.8)	168 (28.0)	167 (27.9)	187 (29.3)
The assessment of the financial situation ****				
Very bad	.	3 (0.5)	8 (1.3)	9 (1.4)
Bad	.	36 (6.0)	46 (7.7)	61 (9.5)
Average	.	276 (46.0)	288 (48.0)	273 (42.7)
Good	.	243 (40.5)	227 (37.8)	262 (41.0)
Very good	.	42 (7.0)	31 (5.2)	34 (5.3)

Population figures are given in thousands. Unless otherwise stated, the population data refer to people aged 18–59. * In Study 1, the study sample was aged between 18 and 54. ** The general population refers to people aged 20–59. *** The structure of the general population refers to the total population of Poland. **** The data refer only to respondents’ declarations, so they do not represent the general population. Source: own elaborations, based on the research [97,98].

**Table 2:** The structure of the samples according to the declared achievement of the subsequent levels (SOC) of sustainable food behaviours.

	Precontemplation	Contemplation	Preparation	Action	Maintenance	Total
Study 1	87 (14.5)	130 (21.7)	159 (26.5)	152 (25.3)	72 (12.0)	100%
Study 2	162 (27.0)	194 (32.3)	109 (18.2)	94 (15.7)	41 (6.8)	100%

Source: own elaboration, based on the research.

**Table 3:** Mapping of measured variables onto COM-B domains.

COM-B Domain	Measured Variable (Study)	Type of Link	Comment
Capability	Food literacy—SFLQ (Study 1)	Indirect proxy	Psychological capability (knowledge, skills) in the food domain; confidence to change is not measured directly.
Opportunity	Awareness of and interest in food-related applications by function (Studies 1–2); cost as the most frequently cited disadvantage (decisional balance)	Indirect proxy	Reflects access to information and practical barriers; physical and social opportunity are not measured.
Motivation	Decisional balance—pros and cons (Studies 1 and 3); value orientations—PVQ-RR (Study 3)	Indirect proxy	Reflective motivation (evaluations, values); automatic motivation is not measured.
Context (outside COM-B)	Food-related well-being—SWFL (Studies 1–2); technology acceptance (Study 2)	Descriptive	Used to describe the groups; not treated as COM-B indicators.

The variables are indirect conceptual proxies for the COM-B domains and do not operationalise them directly. The integrated SOC–COM-B framework has not been validated as a unified measurement instrument.

**Table 4:** Characteristics of respondents in each SOC.

		SOC1/BRG1	SOC2/BRG2	SOC3/BRG3	SOC4/BRG4	SOC5/BRG5
Gender *	Study 1	M: 72.4% F: 27.6%	M: 52.3% F: 47.7%	M: 53.5% F: 46.5%	M: 42.1% F: 57.9%	M: 51.4% F: 48.6%
	Study 2	M: 62.3% F: 37.7%	M: 46.4% F: 53.6%	M: 52.3% F: 47.7%	M: 43.6% F: 56.4%	M: 31.7% F: 68.3%
	Study 3	M: 60.2% F: 39.1% n.a.: 0.7%	M: 56.9% F: 43.1%	M: 43.2% F: 56.1% n.a.: 0.7%	M: 41.0% F: 59.0%	M: 40.9% F: 59.1%
EDU *	Study 1	P + BV: 25.3% S: 46.0% H: 28.7%	P + BV: 13.1% S: 48.5% H: 38.5%	P + BV: 13.2% S: 43.4% H: 43.4%	P + BV: 4.6% S: 44.7% H: 50.7%	P + BV: 5.6% S: 44.4% H: 50.0%
	Study 2	P + BV: 22.8% S: 48.8% H: 28.4%	P + BV: 13.9% S: 50.5% H: 35.6%	P + BV: 11.9% S: 44.0% H: 44.0%	P + BV: 6.4% S: 43.6% H: 50.0%	P + BV: 7.3% S: 39.0% H: 53.7%
Food literacy **	Study 1	2.9 ^a^	3.06 ^ab^	3.22 ^b^	3.46 ^c^	3.64 ^c^
FWB **	Study 1	19.72 ^ab^	18.69 ^a^	19.12 ^ab^	20.22 ^b^	22.94 ^c^
	Study 2	20.65 ^a^	20.14 ^a^	20.74 ^a^	21.62 ^a^	24.00 ^b^

n.a—not available. * χ^2^ test, *p* < 0.001. ** Games–Howell test; *p* < 0.05; the same upper index indicates that these means do not differ in a statistically significant way. Group sizes (n): Study 1—87, 130, 159, 152, 72; Study 2—162, 194, 109, 94, 41 (Table 2); and Study 3—133, 137, 132, 105, 132 for BRG1–BRG5. In Study 3, the columns refer to behavioural-readiness groups (BRG1–BRG5) and not to SOC stages. Source: own elaboration, based on the research.

**Table 5:** Awareness of and interest in food-related mobile applications across SOC groups.

		SOC1	SOC2	SOC3	SOC4	SOC5
Study 1						
Apps that make it easier to choose food products based on their characteristics and ingredients χ^2^ (16, 600) = 130.387, *p* < 0.001, V = 0.233	I don’t know	85.1%	65.4%	45.3%	58.6%	54.2%
	Incl.: I don’t know, but I’d like to use it	26.4%	35.4%	37.1%	45.4%	27.8%
	I know it, but I don’t use it	10.3%	22.3%	34.0%	17.8%	16.7%
	I know it and use it	4.6%	12.3%	20.8%	23.7%	29.2%
Apps that help reduce food waste χ^2^ (16, 600) = 117.945, *p* < 0.001, V = 0.222	I don’t know	78.2%	77.7%	59.1%	71.1%	65.3%
	Incl.: I don’t know, but I’d like to use it	25.3%	45.4%	47.8%	57.9%	34.7%
	I know it, but I don’t use it	18.4%	12.3%	23.3%	17.1%	11.1%
	I know it and use it	3.4%	10.0%	17.6%	11.8%	23.6%
Apps that make it cheaper to buy food χ^2^ (8, 600) = 85.284, *p* < 0.001, V = 0.189	I don’t know	74.7%	62.3%	52.8%	54.6%	52.8%
	Incl.: I don’t know, but I’d like to use it	28.7%	40.8%	40.3%	46.1%	27.8%
	I know it, but I don’t use it	13.8%	20.0%	22.0%	17.1%	20.8%
	I know it and use it	11.5%	17.7%	25.2%	28.3%	26.4%
Study 2						
Apps that make it easier to choose food products based on their characteristics and ingredients χ^2^ (8, 600) = 26.828, *p* < 0.001, V = 0.150	I use and will continue to use	11.1%	16.0%	13.8%	14.9%	14.2%
	I plan to use	48.1%	50.5%	71.6%	59.6%	55.8%
	I don’t use it, and I don’t plan to	40.7%	33.5%	14.7%	25.5%	30.0%
Apps that help reduce food waste χ^2^ (8, 600) = 23.981, *p* = 0.002, V = 0.141	I use and will continue to use	8.6%	14.4%	15.6%	18.1%	13.7%
	I plan to use	48.8%	55.2%	67.9%	54.3%	55.5%
	I don’t use it, and I don’t plan to	42.6%	30.4%	16.5%	27.7%	30.8%
Apps that make it cheaper to buy food χ^2^ (8, 600) = 21.690, *p* = 0.006, V = 0.134	I use and will continue to use	22.2%	22.2%	26.6%	38.3%	24.4%
	I plan to use	45.7%	54.6%	57.8%	44.7%	58.5%
	I don’t use it, and I don’t plan to	32.1%	23.2%	15.6%	17.0%	17.1%

SOC1—precontemplation, SOC2—contemplation, SOC3—preparation, SOC4—action, and SOC5—maintenance. Group sizes (n): Study 1—87, 130, 159, 152, 72; and Study 2—162, 194, 109, 94, 41 (Table 2). Source: own elaboration, based on the research.

**Table 6:** Similarities and differences in the values held by the respondents across the behavioural-readiness groups (BRG).

	BRG1 (n = 133)	BRG2 (n = 137)	BRG3 (n = 132)	BRG4 (n = 105)	BRG5 (n = 132)
Most important values (ranking) *	Societal security (13.99) Self-direction–action (13.73) Benevolence–dependability (13.58)	Societal security (13.81) Self-direction–action (12.91)	Societal security (14.01) Self-direction–action (13.66) Benevolence–dependability (13.38)	Benevolence–dependability (13.76) Societal security (13.64) Self-direction–action (13.60) Self-direction–thought (12.82) Personal security (12.52)	Self-direction–action (13.78) Societal security (13.72) Benevolence–dependability (13.18)
Differentiating values **	Tradition more important than universalism–nature; self-direction–action is as important as personal security and hedonism	Benevolence (dependability and caring) is as important as personal security The above values are more important than hedonism; tradition as important as universalism–nature Hedonism more important than tradition and universalism–nature		Universalism–nature more important than tradition; self-direction–thought more important than personal security	
				Universalism–nature as important as hedonism	Universalism–nature is more important than hedonism

* Friedman’s test, *p* < 0.001. Values are listed in descending order of mean rank, from the most important value down to the last value that does not differ significantly from the top-ranked one (Wilcoxon signed-rank test, *p* > 0.05). ** Based on the Wilcoxon signed-rank test. Values in parentheses are the mean ranks (higher values indicate higher importance). Source: own elaboration, based on the research.

**Table 7:** Design hypotheses derived from SOC- and BRG-specific differences for content and decision-support in sustainable food mobile applications.

Factor	Precontemplation	Contemplation	Preparation	Action	Maintenance
Gender composition patterns	Dominance of male respondents within this SOC suggests the need to consider gender composition when shaping content tone and examples.	Gender composition characteristics relevant for content framing		Dominance of female respondents within this SOC suggests the need to consider gender composition when shaping content tone and examples	
Education and food literacy	Lower content complexity and basic informational framing.	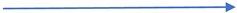		Higher content complexity and more specialised terminology	
Food well-being and motivation	Content framing focused on maintaining perceived food-related well-being while highlighting ancillary benefits.	Content framing focused on increasing FWB			Content framing focused on maintaining FWB
Interest in the application’s functionalities (the higher number of *, the greater the interest)					
Products characteristics and ingredients	**	****	****	*****	**
Food waste prevention	*	****	****	*****	**
Cheaper shopping	**	****	****	*****	**
Value-aligned content framing considerations.	Independence in decision-making and achieving chosen goals. Social security.				
	Pleasure. Prestige.	Caring for others. Prestige. Pleasure.	Caring for others. Prestige. Pleasure.	Being reliable caring for others. Striving for justice. Ecology. Pleasure.	Being reliable. Ecology. Pleasure.
Content themes potentially misaligned with dominant value orientations.	Ecology. Novelty.	Ecology. Novelty. Tradition.	Novelty.	Novelty.	Novelty. Tradition.
Shared content anchors across SOCs.	Consistent importance of health-related considerations. Low relevance of trend- or fashion-driven sustainability cues.				
Stage-specific content emphasis.	Predominant importance of health-related considerations.		Increasing relevance of local economy support considerations alongside health.	The broader importance of health, ecological and local economy support considerations.	
Addressing the most common barriers (which are common to all SOCs).	Recognition of consumer autonomy in food choices, with lower receptiveness to prescriptive or normative sustainability framing. Lower effectiveness of persuasion-based messaging and imposed dietary narratives. Higher importance of discounted shopping applications as an initial point of engagement, with additional supportive features gaining relevance at later stages. Relevance of price comparison tools in reducing perceived economic barriers.				
Addressing the stage-specific perceived barriers.	Lower perceived difficulty through familiarisation with available options and supportive features addressing common practical concerns. Support for product discovery, shopping planning, and in-store localisation at later SOC stages.			Higher relevance of clear information about product availability and points of purchase. Increased importance of decision-support features facilitating shopping planning and meal preparation. Importance of product discovery and in-store localisation support in reducing practical barriers at later SOC stages.	

Note: The table does not report consumer preferences or tested communication effectiveness. The entries are design hypotheses (level 3 of evidence) derived from the observed group differences in Table 4, Table 5 and Table 6; they have not been tested. For Study 3, the columns correspond to behavioural-readiness groups (BRG). Source: own elaboration, based on the research.

**Table 8:** The SOC–COM-B framework: matching users’ behavioural readiness with content guidelines and application features for sustainable food consumption.

COM-B Component	Precontemplation	Contemplation	Preparation	Action	Maintenance
Capability characteristic	• Low food literacy • Need for low content complexity and basic informational framing	• Gradual capability building through progressive knowledge exposure • Emergence of goal-related information needs	• Need for personalised, practice-oriented guidance	• High relevance of tools supporting behaviour execution (e.g., shopping and meal-planning aids)	• Reinforcement of acquired knowledge • Preference for more advanced, efficiency-oriented information
Opportunity characteristic	• Limited interest in applications • Low awareness of available tools	• High interest across functional categories (product information, food-waste reduction, price-related tools)	• Continued high interest in diverse application functionalities	• Peak relevance of application support across product, waste, and price domains	• Reduced need for functional support • Selective use of applications for maintenance purposes
Motivation characteristic	Dominant values: independence, social security, pleasure Main motivator: health Content themes potentially misaligned: ecology, novelty	Dominant values: caring for others, prestige, pleasure Main motivator: health Content themes potentially misaligned: ecology, novelty, tradition	Dominant values: caring for others, prestige, pleasure Main motivators: health and support for the local economy	Dominant values: reliability, caring for others, justice, ecology, pleasure Main motivators: health, ecology, local economy	Dominant values: reliability, ecology, pleasure Main motivators: health and ecology
**Support-over-persuasion communication logic**	• Emphasis on autonomy and freedom of choice • Avoidance of prescriptive or normative framing • Framing sustainable practices as simple and non-disruptive	• Reinforcement of voluntary engagement • Framing applications as supportive rather than directive • Use of gentle, accessible suggestions	• Framing applications as facilitators of daily routines (e.g., planning, time efficiency) • Avoidance of moralising tone	• Emphasis on practical assistance at decision points (e.g., locating products, simplifying shopping processes) • Absence of behavioural pressure	• Focus on continuity and maintenance support • Use of soft reminders rather than prompts aimed at change • Tools supporting behavioural stability rather than initiation

Note: The framework provides a conceptual structure for matching digital content and functional support with consumers’ behavioural readiness across stages of change. It does not recommend specific content elements or predict behavioural outcomes. Source: own elaboration.

## Data Availability

The datasets analysed during the current study are available from the corresponding author upon request.

## Supplementary Materials

The following supporting information can be downloaded at: https://www.mdpi.com/article/10.3390/nu18213707/s1, Table S1: Characteristics of respondents in each SOC; Table S2: Ranking of values within BRG groups (average ranks according to the Friedman test); Table S3: The Wilcoxon signed-rank test—the most important values in BRG groups; Table S4: Decision balance—comparison of means.
