# Supplementary material for: The SOC–COM-B Framework for Sustainable Food Choices: Tailoring Mobile Application Content to Consumers’ Behavioural Readiness
Source: Nutrients. 2026 Nov 8;18(21):3707. doi: 10.3390/nu18213707 (PMC13765468; doi:10.3390/nu18213707)
Supplement: Supplementary file 1 [file nutrients-18-03707-s001.zip › nutrients-4626443-supplementary.pdf]

Supplementary Materials

For data that met the assumption of homogeneity of variance (based on Levene's test), we applied Hochberg's GT2 post-hoc test, while for data that did not meet this assumption, we used the Games-Howell test.

Table S1. Characteristics of respondents in each SOC

		SOC1/ BRG1	SOC2/ BRG2	SOC3/ BRG3	SOC4/ BRG4	SOC5/ BRG5
Gender	Study 1* ($p<0.001$, $V=0.185$)					
	M	72.4%	52.3%	53.5%	42.1%	51.4%
	F	27.6%	47.7%	46.5%	57.9%	48.6%
	Study 2* ($p<0.001$, $V=0.174$)					
	M	62.3%	46.4%	52.3%	43.6%	31.7%
	F	37.7%	53.6%	47.7%	56.4%	68.3%
	Study 3* ($p=0.007$, $V=0.129$)					
	M	60.2%	56.9%	43.2%	41.0%	40.9%
	F	39.1%	43.1%	56.1%	59.0%	59.1%
	n.a.	0.7%		0.7%		
Age	Study 1*($p=0.207$)					
	18-24	12.6%	21.5%	21.4%	15.8%	11.1%
	25-34	34.5%	33.1%	28.3%	32.9%	41.7%
	35-44	31.0%	24.6%	34.6%	34.9%	23.6%
	45-54	21.8%	20.8%	15.7%	16.4%	23.6%
	Study 2* ($p=0.231$)					
	18-29	16.7%	27.8%	30.3%	23.4%	12.2%
	30-39	30.2%	28.4%	22.0%	29.8%	29.3%
	40-49	27.2%	24.2%	28.4%	28.7%	34.1%
	50-59	25.9%	19.6%	19.3%	18.1%	24.4%
	Study 3* ($p=0.059$)					
	18-29	25.6%	25.5%	22.0%	19.0%	12.9%
	30-39	32.3%	32.1%	28.8%	27.6%	25.8%
	40-49	24.1%	24.1%	32.6%	24.8%	34.1%
	50-59	18.0%	18.2%	16.7%	28.6%	27.3%
EDU	Study 1* ($p<0.001$, $V=0.161$)					
	P+BV	25.3%	13.1%	13.2%	4.6%	5.6%
	S	46.0%	48.5%	43.4%	44.7%	44.4%
	H	28.7%	38.5%	43.4%	50.7%	50.0%
	Study 2* ($p<0.001$, $V=0.150$)					
	P+BV	22.8%	13.9%	11.9%	6.4%	7.3%
	S	48.8%	50.5%	44.0%	43.6%	39.0%
	H	28.4%	35.6%	44.0%	50.0%	53.7%
	Study 3* ($p=0.729$)					
	P+BV	9.8%	11.7%	9.1%	7.6%	10.6%
	S	41.4%	38.0%	39.4%	34.3%	31.8%
	H	48.9%	50.4%	51.5%	58.1%	57.6%
Place of residence	Study 1* ($p=0.242$)					
	A	49.4%	43.1%	42.8%	34.2%	27.8%
	B	12.6%	13.8%	13.2%	12.5%	12.5%
	C	18.4%	17.7%	17.6%	16.4%	31.9%
	D	13.8%	15.4%	14.5%	21.0%	16.6%
	E	5.7%	10.0%	11.9%	15.8%	11.1%

	Study 2* (p=0.085)					
	A	46.9%	38.7%	41.3%	35.1%	26.8%
	B	15.4%	11.3%	12.8%	11.7%	14.6%
	C	14.2%	20.6%	18.3%	20.2%	31.7%
	D	11.7%	13.9%	20.2%	24.5%	14.6%
	E	11.7%	15.5%	7.3%	8.5%	12.2%
	Study 3* (p=0.062)					
	A	44.4%	36.5%	36.4%	35.2%	40.2%
	B	8.3%	19.0%	12.1%	16.2%	9.1%
	C	13.5%	21.9%	24.2%	15.2%	20.5%
	D	15.0%	12.4%	15.2%	21.9%	19.7%
	E	18.8%	10.2%	12.1%	11.4%	10.6%
The assessment of the financial situation	Study 1* (p=0.483)					
	very bad/bad	8.0%	7.7%	7.5%	5.3%	2.8%
	average	47.1%	44.6%	51.6%	44.1%	38.9%
	good	36.8%	40.8%	35.8%	44.7%	45.8%
	very good	8.0%	6.9%	5.0%	5.9%	12.5%
	Study 2* (p=0.404)					
	very bad/bad	9.3%	11.8%	4.6%	9.6%	4.9%
	average	51.9%	47.9%	46.8%	47.9%	36.6%
	good	32.7%	38.1%	42.2%	35.1%	51.2%
	very good	6.2%	2.1%	6.4%	7.4%	7.3%
	Study 3* (p=0.231)					
	very bad/bad	14.3%	8.0%	13.6%	11.4%	7.6%
	average	48.9%	44.5%	37.1%	46.7%	37.1%
	good	32.3%	43.1%	44.7%	36.2%	47.7%
	very good	4.5%	4.4%	4.5%	5.7%	7.6%
Food literacy	Study 1** (η^2=0.193)	2.9^{a}	$3.06^{a,b}$	3.22^{b}	3.46^{c}	3.64^{c}
FWB	Study 1** (η^2=0.084)	19.72^{ab}	18.69^{a}	19.12^{ab}	20.22^{b}	22.94^{c}
	Study 2** (η^2=0.042)	20.65^{a}	20.14^{a}	20.74^{a}	21.62^{a}	24.00^{b}
General technology acceptance	Study 2* (p=0.363)					
	I like to be the first person to buy new products when they go on sale.	15.4%	15.5%	18.3%	18.1%	17.1%
	I often buy things straight away, but I do think about it a little first.	35.2%	40.7%	45.9%	27.7%	34.1%
	I only buy it after some of my friends have tried it.	26.5%	26.3%	24.8%	31.9%	26.8%

I buy something when most of my friends have already bought it and given it a positive review	14.8%	12.4%	10.1%	17.0%	19.5%
I don't really want to buy it.	8.0%	5.2%	0.9%	5.3%	2.4%

* Testing the significance of differences using the chi-squared test. V – Cramer's V – an effect size calculated when the differences between groups are statistically significant.

** Games-Howell test; $p<0.05$; The same upper index indicates that these means do not differ in a statistically significant way. η^2 – effect size.

Group sizes (n): Study 1 – 87, 130, 159, 152, 72; Study 2 – 162, 194, 109, 94, 41 (Table 2); Study 3 – 133, 137, 132, 105, 132 for BRG1–BRG5. In Study 3, the columns refer to behavioural-readiness groups (BRG1–BRG5) and not to SOC stages.

EDU: P – primary; BV – basic vocational; S – secondary; H – higher

Place of residence: A- village, B- city up to 20,000 inhabitants, C-city from 20,000 to 100,000 inhabitants, D-city from 100,000 to 500,000 inhabitants, E – city over 500,000 inhabitants

Source: own elaboration based on the research

Table S2. Ranking of values within BRG groups (average ranks according to the Friedman test)

Value	BRG1	BRG2	BRG3	BRG4	BRG5
achievement	8.68	9.10	8.61	8.23	8.12
hedonism	12.18	10.65	11.34	10.86	11.16
stimulation	6.50	7.01	6.24	6.63	6.32
self-direction-action	13.73	12.91	13.66	13.60	13.78
self-direction-thought	12.67	12.21	12.65	12.82	12.71
universalism-tolerance	9.64	10.30	9.96	10.56	11.09
universalism-nature	6.78	8.56	9.59	10.73	12.35
universalism-societal concern	10.78	11.18	11.31	11.74	11.92
benevolence-caring	12.62	12.29	12.71	12.57	12.14
benevolence-dependability	13.58	12.59	13.38	13.76	13.18
humility	7.76	7.85	6.67	7.39	7.19
conformity-interpersonal	8.19	8.58	7.70	7.95	8.02
conformity-rules	8.96	9.03	9.16	10.08	9.27
tradition	9.32	8.64	8.83	8.11	8.21
security-societal	13.99	13.81	14.01	13.64	13.72
security-personal	12.64	12.63	12.96	12.52	12.11
face	11.00	10.93	11.67	10.55	10.42
power-resources	6.94	7.07	6.00	5.18	4.92
power-dominance	4.05	4.66	3.55	3.08	3.38
N	133	137	132	105	132
χ^2 (18)	659.058	551.439	725.741	607.599	822.722
p	<0.001	<0.001	<0.001	<0.001	<0.001

Source: own elaboration based on the research

Table S3. The Wilcoxon signed-rank test – the most important values in BRG groups

**BRG1**	security -societal				
	self-direction- action	benevolence-dependability	self-direction-thought		
Z	-0.278[a]	-1.283[b]	-2.063[b]		
p	0.781	0.199	0.039		
**BRG2**	security -societal				
	self-direction- action	benevolence-dependability			
Z	-1.870[b]	-2.888[b]			
p	0.061	0.004			
**BRG3**	security -societal				
	self-direction- action	benevolence-dependability	self-direction-thought		
Z	-0.838[b]	-1.622[b]	-2.79[b]		
p	0.402	0.105	0.023		
**BRG4**	benevolence-dependability				
	security - societal	self-direction- action	self-direction- thought	security - personal	benevolence- caring
Z	-0.303[b]	-0.577[a]	-1.294[b]	-1.891[b]	-2.513[b]
p	0.762	0.564	0.195	0.059	0.012
**BRG5**	self – direction - action				
	Security - societal	benevolence - dependability	self-direction-thought		
Z	-0.147[b]	-1.728[b]	-2.945[b]		
p	0.883	0.084	0.003		

a – based on negative ranks

b – based on positive ranks

Source: own elaboration based on the research

Table S4. Decisional balance – comparison of means

PROs/CONs	η^2	SOC1/ BRG1	SOC2/ BRG2	SOC3/ BRG3	SOC 4/ BRG4	SOC5/ BRG5
Study 1		n=87	n=130	n=159	n=152	n=72
planet protection***	0.131	3.15^{a}	3.53^{b}	3.77^{b}	4.16^{c}	4.13^{c}
better for my health***	0.147	3.67^{a}	3.96^{a}	3.96^{a}	4.61^{b}	4.54^{b}
better quality diet***	0.168	3.55^{a}	3.87^{b}	4.03^{b}	4.52^{c}	4.54^{c}
support for the local economy***	0.064	3.49^{a}	3.58^{a}	3.79^{ab}	4.13^{b}	4.06^{b}
proud to be helping the environment***	0.087	3.28^{a}	3.55^{ab}	3.84^{bc}	4.10^{c}	4.01^{c}
impractical***	0.037	3.14^{ab}	3.29^{ab}	3.43^{b}	2.94^{a}	2.94^{a}
too expensive***	0.046	3.34^{ab}	3.79^{c}	3.56^{bc}	3.41^{ab}	3.07^{a}
too difficult***	0.074	3.01^{b}	3.52^{c}	3.28^{bc}	2.94^{ab}	2.61^{a}
product unavailability**	0.026	3.06^{ab}	3.38^{b}	3.26^{b}	3.04^{ab}	2.85^{a}
unable to find it at the shop**	0.027	2.99^{ab}	3.29^{b}	3.34^{b}	3.12^{ab}	2.81a
Study 3		n=133	n=137	n=132	n=105	n=132
planet protection***	0.236	2.85^{a}	3.38^{b}	3.82^{c}	4.10^{cd}	4.36^{d}
better for my health***	0.157	3.45^{a}	3.86^{b}	4.39^{c}	4.35^{c}	4.48^{c}
better quality diet***	0.179	3.38^{a}	3.76^{b}	4.22^{c}	4.30^{c}	4.52^{c}
support for the local economy***	0.199	3.15^{a}	3.62^{b}	4.11^{c}	4.10^{c}	4.47^{d}
proud to be helping the environment***	0.235	2.83^{a}	3.42^{b}	3.85^{c}	4.08^{cd}	4.37^{d}
keeping up with trends***	0.124	2.16^{a}	3.04^{b}	3.02^{b}	3.10^{b}	3.55^{c}
other people who are important to me***	0.127	2.05^{a}	2.76^{b}	2.65^{b}	3.05^{bc}	3.39^{c}
impossible right now in my life	0.009	2.98	3.02	3.03	2.99	2.75
too expensive	0.010	3.68	3.58	3.71	3.65	3.42
too difficult**	0.021	3.43^{a}	3.34^{ab}	3.28^{ab}	3.13^{ab}	3.01^{b}
product unavailability	0.010	2.85	3.05	3.01	3.15	2.91
unable to find it at the shop	0.013	2.87	3.05	3.03	3.21	2.88
the right to eat whatever you like, regardless of what others think	0.009	3.99	3.78	4.05	3.91	3.88
contrary to Polish tradition*	0.017	2.56^{ab}	2.91^{b}	2.58^{ab}	2.58^{ab}	2.45^{a}

ANOVA: *** $p<0.001$, ** $p<0.01$, * $p<0.05$; η^2 – effect size.

Games-Howell test; $p<0.05$; The same upper index indicates that these means do not differ in a statistically significant way.

Source: own elaboration based on the research
